# VULNERABILITY TO ANTICHOLINERGIC MEDICATION ASSOCIATED FRAILTY IN LOW-INCOME MINORITY OLDER ADULTS

**DOI:** 10.1093/geroni/igac059.2126

**Published:** 2022-12-20

**Authors:** Lana Sargent, Matthew Barrett, Huma Nawaz, Marissa Mackiewicz, Youssef Roman, Patricia Slattum, Sarah Hobgood, Elvin Price

**Affiliations:** VCU School of Nursing, Ashland, Virginia, United States; Virginia Commonwealth University, Richmond, Virginia, United States; Virginia Commonwealth University, Richmond, Virginia, United States; Virginia Commonwealth University, Richmond, Virginia, United States; Virginia Commonwealth University, Richmond, Virginia, United States; Virginia Commonwealth University, Richmond, Virginia, United States; Virginia Commonwealth University, Richmond, Virginia, United States; Virginia Commonwealth University, Richmond, Virginia, United States

## Abstract

Low-income minority older adults are highly susceptible to drug adverse effects of medications due to aging, comorbidities, and polypharmacy. Several studies have demonstrated anticholinergic medication is associated with frailty, supporting the hypothesis for mechanistic peripheral nervous system effects. The goal of this cohort study is to determine peripheral nervous system effects of anticholinergic medication exposure with frailty by conducting a sensitivity analysis using multiple anticholinergic tools. Spearman correlation matrix and intraclass correlation coefficients (ICC) are used to determine the function of five clinical Anticholinergic Burden Scales (ACBS): Anticholinergic Burden Scale (ACB), Anticholinergic Drug Scale (ADS), total standardized daily doses (TSDD), and Cumulative Anticholinergic Burden scale (CAB). Ordinal logistic regression and area under the curve (AUC) are used to evaluate anticholinergic burden-associated frailty models. The cohort included 80 individuals (mean age = 69 years; 55.7% female, 71% African American). Among individuals prescribed anticholinergics, 33% are robust, 44% pre-frail, and 23% frail. All scales are highly correlated with each other (p < 0.001), ICC3 = 0.66 (p < 0.001, CI 95% 0.53-0.73). All five of the scales predicted pre-frail and frail status (p < 0.05) with low misclassification rates for frail individuals (AUC = 0.70 – 0.80). Considering ACBS are highly correlated and all predict frailty; all of the scales can be used in future frailty research; however, the CAB and TSDD consider both potency and dose. Additional research is necessary to understand the peripheral nervous system effects of anticholinergic drug exposure and if deprescribing can improve frailty status.

